# Assessing the Role of Socio-Demographic Triggers on Kolmogorov-Based Complexity in Spoken English Varieties

**DOI:** 10.3390/e27101009

**Published:** 2025-09-26

**Authors:** Katharina Ehret

**Affiliations:** Department of English, University of Freiburg, Rempartstr. 15, 79089 Freiburg, Germany; katharina.ehret@anglistik.uni-freiburg.de

**Keywords:** language complexity, Kolmogorov complexity, English varieties, language contact, typology, corpus linguistics

## Abstract

This paper assesses the role of socio-demographic triggers on Kolmogorov-based complexity in spoken English varieties. It thus contributes to the ongoing debate on contact and complexity in the sociolinguistic typological research community. Currently, evidence on whether socio-demographic triggers influence the morphosyntactic complexity of languages is controversial and inconclusive. Particularly controversial is the influence of the proportion of non-native speakers and the number of native speakers, which are both common proxies for language contact. In order to illuminate the issue from an English-varieties perspective, I use regression analysis to test several socio-demographic triggers in a corpus database of spoken English varieties. Language complexity here is operationalised in terms of Kolmogorov-based morphological and syntactic complexity. The results only partially support the idea that socio-demographic triggers influence morphosyntactic complexity in English varieties, i.e., speaker-related triggers turn out to be negative but non-significant. Yet, net migration rate shows a positive significant effect on morphological complexity which needs to be seen in the global context of English as a commodity and unequal access to English. I thus argue that socioeconomic triggers are better predictors for complexity than demographic speaker numbers. In sum, the paper opens up new horizons for research on language complexity.

## 1. Introduction

This paper marries quantitative corpus-based linguistics with information theory and explores the relationship between socio-demographic triggers and Kolmogorov-based language complexity in an English-varieties context. Language complexity has been a widely researched and, at times, hotly debated topic in the sociolinguistic typological community for the past two decades [1,2,3,4]. The pivotal point of the original complexity debate was the question of whether all languages were, overall, equally complex or not [5]. Thus, much linguistic research on complexity focuses on how to define [4,6] and measure language complexity [7,8], or how to evaluate existing complexity metrics [9]. It is fair to say that language complexity has become a well-researched and measurable aspect of language variation, with a substantial body of evidence suggesting that languages and language varieties can and do differ in their complexity in various linguistic subdomains [10,11,12]. The major question currently under debate in the sociolinguistic typological complexity community is the degree to which observed variation in language complexity can be attributed to socio-demographic triggers such as language contact or the status of a language in a society (e.g., vitality, official status). In particular, language contact prominently features as a key explanatory trigger in the literature [13,14,15,16]. This line of research dates back to contact scenarios described in [17] and a paper by Wray and Grace, which connects community structure to linguistic structure [18]. Essentially, languages used for communication in small, isolated speech communities with close social networks (*esoteric* communication) tend to maintain or foster comparatively more complex structures [17,18]. In contrast, languages used by comparatively larger speech communities as well as for communication between different communities (*exoteric* communication) tend towards simplification [17,18]. This hypothesis, which has become known as the *Linguistic Niche Hypothesis* [16], has been extensively tested in a number of large-scale typological studies. However, empirical evidence on the influence of language contact, often but not exclusively approximated in terms of the number of native speakers [16] or the proportion of non-native speakers [15], is controversial and inconclusive. While some large-scale typological studies report significant effects of language contact and exotericity on various morphosyntactic structures [15,16,19], other studies find no such effects in their data [12,20,21].

Against this backdrop, I assess the influence of several socio-demographic triggers related to language contact and exotericity including, inter alia, well-known triggers such as the number of native speakers and the proportion of non-native speakers. Furthermore, this paper is the first, to my knowledge, to feature net migration rate as a trigger for mobility and thus language contact. These triggers are tested in a geographically and typologically representative text database of spoken English varieties which consists of several well-known English corpora. Language complexity is measured in terms of an information-theoretic, Kolmogorov-based complexity measure [10,22], which is applied to assess the morphological and syntactic complexity of the text samples. Subsequently, state-of-the-art regression analysis is used to assess the impact of socio-demographic triggers on Kolmogorov-based complexity—henceforth simply complexity—in spoken English varieties. It is expected that all triggers have negative effects on complexity. The results, however, show no significant effects of speaker-related triggers on morphological complexity when controlling for corpus and geography. However, the observed effect directions are theoretically interesting as they are, indeed, negative. Furthermore, there are no effects for contact languages, but population density and net migration rate show some significant effect on morphological complexity. An important corollary of the observed effect directions is that net migration rate is primarily a socioeconomic trigger rather than a predictor for contact. Overall, I argue that socioeconomic triggers might be a better predictor for complexity in general, and English varieties in particular, than demographic speaker data.

In short, this paper provides a fresh perspective on contact and complexity by (1) taking a corpus- and thus usage-based approach, and (2) testing the Linguistic Niche Hypothesis in an English-varieties context. Following the Freiburg tradition of complexity research [22,23,24,25], I thus explore cross-linguistic hypotheses in the comparatively well-charted territory of intra-language complexity variation in English. At the same time, the paper highlights and discusses the applicability of Kolmogorov complexity as a measure of language complexity.

## 2. Materials and Methods

All numeric data discussed in this section is openly available at GitHub (https://github.com/Morphosyntactic-Variation-in-Englishes/k-complexity, accessed on 11 August 2025). The demographic materials including original sources and definitions were taken from https://github.com/Morphosyntactic-Variation-in-Englishes/DOVE (accessed on 11 August 2025). Unless otherwise stated, all analyses were conducted in *R v4.5.1* [26].

### 2.1. Corpora and Socio-Demographic Data

To furnish a case study, a typologically and geographically representative text database of English varieties is compiled sampling orthographically transcribed spoken texts from three well-known English corpora: the *International Corpus of English* (ICE [27]) for indigenised second-language (L2) and high-contact first-language (L1) varieties around the world, the *Freiburg Corpus of English Dialects* (FRED [28]) for low-contact and high-contact L1 varieties in the British Isles, and the *Santa Barbara Corpus of Spoken American English* (SBCSAE [29]) for spoken American English. ICE is a synchronic corpus collection sampling spoken and written registers of national varieties of English around the world. The analysis here is restricted to texts labelled as S1A in ICE because these comprise spoken face-to-face conversations which are of primary interest in this paper. In contrast, FRED is the most extensive corpus of traditional spoken varieties (also known as dialects) in the British Isles and is based on oral history interviews. The SBCSAE comprises spoken interactions which primarily consist of face-to-face conversations but also include other spoken language use (e.g., card games or story-telling). For the purposes of this paper, the FRED and SBCSAE data are for now considered sufficiently close to face-to-face conversation and hence roughly comparable to the spoken data in ICE. (Subsequent inspection of boxplots revealed that the entire SBCSAE is an outlier. Thus, the corpus was excluded from further analyses.)

The typological classification of English varieties follows the classification in the *electronic World Atlas of Varieties of English* v3.0 (eWAVE [30]). eWAVE distinguishes three language types which were constructed on the basis of the socio-historical background and (historic) language contact situation of the individual English varieties. Consequently, this classification needs to be considered a theoretical construct and does not correspond to common typological classifications which are mostly based on language structure or genealogy [31]. In short, high-contact indigenised L2 varieties comprise varieties mainly in former colonies of the British empire (e.g., Indian English, Ugandan English) [30]. Note that these varieties are labelled as non-native varieties in eWAVE despite the fact that most indigenised L2 varieties are (increasingly) spoken as native varieties (often as part of speakers’ multilingual repertoires). High-contact L1 varieties comprise English varieties which have been in contact with either other English varieties or other languages during their history. This type includes varieties in former settlement colonies (e.g., New Zealand English), language-shift varieties such as Irish English, and standard L1 varieties (e.g., Canadian English). Finally, low-contact varieties comprise regional non-standard varieties (like Scottish English) which have long since been established as mother tongue varieties and have been comparatively isolated in their recent history [30]. In the context of the Linguistic Niche Hypothesis, high-contact L1 and indigenised L2 varieties roughly correspond to exoteric languages, while low-contact L1 varieties roughly correspond to esoteric languages. In total, the final database covers 24 varieties, specifically, 9 high-contact L1 varieties, 9 indigenised L2 varieties, and 6 low-contact varieties.

All socio-demographic data was taken from *A socio-demographic Dataset fOr Varieties of English* v1.0 (DOVE [32]) which was specifically compiled to investigate the relationship between language complexity and language external triggers in varieties of English. DOVE draws on numerous open-access sources, mainly census data or non-governmental surveys for speaker numbers, but also builds on information from eWAVE [30]. Currently, the database comprises information on six socio-demographic triggers for 25 English varieties [32]. The socio-demographic triggers analysed in this paper are the proportion of non-native speakers, the number of native speakers, the geographic spread of the variety, population density, net migration rate per country, and all contact languages spoken in a country. Note that the numbers reported for these triggers comprise only the numbers of speakers/languages reported for individual countries or territories in which the variety is primarily spoken, thus excluding, e.g., diaspora speakers [32]. On a side note, the terms native and non-native speaker are adopted in this paper as is customary in the sociolinguistic–typological complexity literature. I explicitly acknowledge and point out that these concepts are inherently problematic [33], especially in an English-varieties context [34]. Thus, I emphasise that no (d)evaluative judgements regarding proficiency, identity, or ethnicity of speakers are made in using these terms.

On a conceptual plane, non-native speakers are defined as adult second-language learners, following [14,15]. Thus, it is assumed that large-scale (adult) second-language acquisition and the accommodation of adult second-language learners by native speakers lead to the simplification of morphosyntax as suggested by iterated learning experiments [35,36] and psycholinguistic experimental set-ups [37]. The proportion of non-native speakers is calculated as the proportion of non-native speakers in the total speaker population whereby the total population comprises both native and non-native speakers [15]. The number of native speakers can also be used to approximate language contact as non-native acquisition because languages spoken by larger numbers of native speaker communities, i.e., exoteric communities, are also frequently acquired and used by outsiders [16]. Net migration rate per country and population density are newcomers among the socio-demographic triggers customarily analysed. Yet, net migration rate can be taken as a proxy both for language contact in the sense that comparatively higher net migration rates indicate increased language contact with outsiders, and as a proxy for mobility. Mobility, like population density, is associated with exotericity and has recently been found to correlate with morphosyntactic structures when analysed together with other socio-demographic triggers [38]. Population density is calculated as the total speaker population divided by the geographic spread of a given variety. Finally, the number of languages spoken in specific territories or neighbouring these territories are also frequently analysed as a proxy for language contact [15,16,21]. In this paper, languages are operationalised as all contact languages spoken in a given country, which are spoken by at least ten percent of the total population in this country. The rationale is that structural borrowing or source language interference on the level of grammar only occurs when a target language is acquired by a substantial number of non-native speakers [39].

Although the influence of geography on morphosyntax in English varieties has been reported to be rather weak [40,41,42], region is included to control for potential geographic autocorrelation. The English varieties in this dataset are located in the following six geographic macro-regions: Africa (Af), the Americas (Am), Asia (As), Oceania (Oc), Caribbean (Ca), and the British Isles (BI) [32].

### 2.2. Kolmogorov Complexity as a Measure of Language Complexity

Kolmogorov complexity [43,44] was first applied in linguistics as a measure of language complexity by [45,46]. It has since been extended and adapted for use with naturalistic, non-parallel language corpora [10,22]. Although Kolmogorov complexity is, for mathematical reasons related to the halting problem, uncomputable, its upper bounds can be approximated by using entropy estimation methods [43,47]. Conveniently, modern off-the-shelf compression programs like *gzip* employ a variant of entropy estimation that approximates Kolmogorov complexity [48,49]. In this spirit, compression programs are used to measure language complexity in texts, whereby the complexity of a given text is thereby defined as the length of the shortest possible description of this text from which the original text can be reconstructed [48] (p. 3252). The text strings below illustrate how language complexity is estimated by approximating Kolmogorov complexity: both original text strings have a length of 10 symbols. Yet, the first string can be compressed to a length of four symbols, whereas the shortest possible description of the second string is the string itself. In terms of Kolmogorov complexity, the first string is considered less complex than the second string.

(1) kckckckckc→5×kc

(2) kc?plmn2e!→kc?plmn2e!

Kolmogorov complexity as a measure of language complexity in this paper is implemented with the compression technique [10,50] and utilised to measure complexity at the morphological and syntactic level. The compression technique employs the open-source algorithm *gzip* (v1.12) for text compression. As correctly noted by a reviewer, *gzip* is not necessarily the most efficient compression algorithm for finite data. For the current purpose of calculating and comparing compression ratios, however, efficiency does not matter. The scripts for implementation are available at GitHub (https://github.com/katehret/measuring-language-complexity, accessed on 29 May 2025).

Complexity at the morphological and syntactic level is indirectly measured through modification of the morphological and syntactic information in texts prior to compression. Specifically, the morphological and syntactic information is modified by random deletion, as described in [10]. Morphological modification is achieved by deleting 10% of all orthographically transcribed characters in a text, thus creating more word form variation. Morphologically complex languages, i.e., languages with a high number of different word forms, are less affected by this procedure than morphologically less complex languages. Syntactic modification is achieved by deleting 10% of all word tokens in a text, thus disrupting word order patterns. This procedure mainly affects languages with many word order rules and rigid word order [10]. The morphological and syntactic complexity scores obtained with the compression technique essentially indicate how well the compression algorithm deals with the noise created through the morphological and syntactic text modification, respectively. For more detailed descriptions of corpus preparation and implementation refer to the instructions on GitHub and [10,50].

Technically, two measures per text are taken: the compressed file size of the original unmodified text (defined as *c*) and the compressed file size of the modified texts, whereby the morphologically modified text is defined as *m* and the syntactically modified text is defined as *s*. Based on these measures, a morphological complexity ratio

(3) −m/c,

and a syntactic complexity ratio

(4) s/c

are calculated for each text.

In naturalistic corpora, the compression technique is implemented iteratively with n=1000 iterations over equally sized random samples of the texts for reasons of comparability and content control [10] (p. 10). Thus, the compression technique returns mean morphological and syntactic complexity ratios—henceforth simply complexity ratios—for each text [10].

In terms of interpretation, morphological complexity is a measure of word form variation. To be more precise, it conflates some traditional morphological complexity measures related to structural word form regularity (including inflectional and derivational complexity), and lexical diversity [10] (p. 8).Thus, comparatively higher compression ratios (i.e., ratios closer to 0) indicate comparatively more morphological complexity. In other words, texts with comparatively higher morphological compression ratios are marked by more word form variation and hence higher morphological complexity. Syntactic complexity, on the contrary, is a measure of structural word order rigidity and word order rules. Thus, relatively lower syntactic compression ratios indicate less word order rigidity and less syntactic complexity. In contrast, comparatively higher compression ratios indicate more rigid word order and more syntactic complexity [10] (pp. 16–17). It needs to be stressed, however, that both measures are inherently unsupervised. Kolmogorov-based complexity measures are holistic, usage-based measures of structural surface redundancy and irregularity. This means that they are largely agnostic about deeper linguistic form–function pairings. For details and examples of the type of complexity captured by compression programs of the Lempel–Ziv family refer to [50].

### 2.3. Statistical Methods

The role of socio-demographic triggers on complexity in spoken English varieties is assessed using linear regression, implemented with the *lme4* library [51]. Two theoretically informed models are thus constructed: one model featuring morphological complexity and one featuring syntactic complexity as response variable. The impact of the socio-demographic triggers is assessed at the level of variety (rather than individual text files) because, on the one hand, the data is available at the level of varieties. On the other hand, when attempting to model socio-demographic data with file-level observations, I encountered convergence and statistical issues, presumably because the socio-demographic data was identical across multiple observations, or because of the very small scale and differences among the individual complexity ratios, or possibly both. Thus, the complexity ratios are aggregated by variety for modelling. Furthermore, scaling and centering is applied to the aggregate complexity ratios to make them more commensurable and aid model convergence.

Prior to modelling, all numeric socio-demographic triggers are scaled and centered, and the number of native speakers is log-transformed using the base-10 logarithm [14]. Contact languages is converted to a factor with three levels as its values range only from 0 to 3. The proportion of non-native speakers is not transformed. These triggers are included as fixed effects in the models. In order to control for potential geographic effects and effects of corpus, both macro-region and corpus are included as varying intercepts.

The model for morphological complexity converges without warnings and does not violate assumptions of normality (p=0.733) and heteroscedasticity (p=0.744). All variance inflation factors are <3.0.

**Figure 1:**
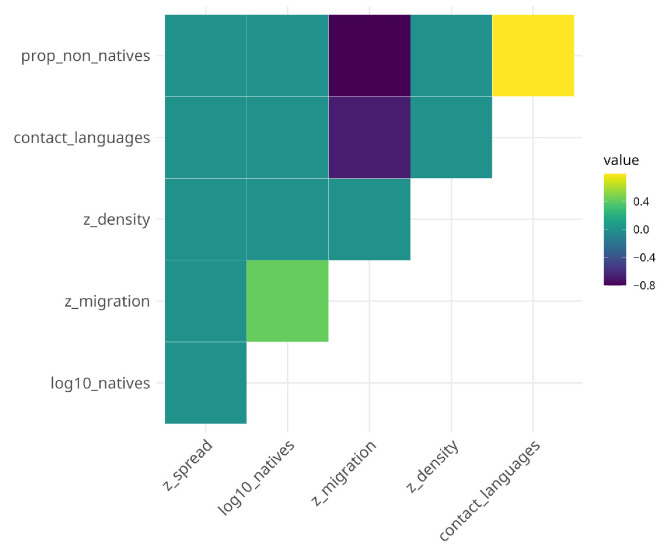
Correlation plot of socio-demographic triggers.

In contrast, the full model for syntactic complexity converges with a singular fit. Initial model inspection furthermore revealed harmful collinearity for the trigger contact languages indicated by a variance inflation factor of VIF=13.14 and moderate correlation for proportion of non-native speakers (VIF=5.33). Collinearity is addressed by removing the number of contact languages from the model but retaining the proportion of non-native speakers. This is justified as both triggers are highly positively correlated (*r* = 0.79, p<0.001; see also Figure 1).

**Figure 2:**
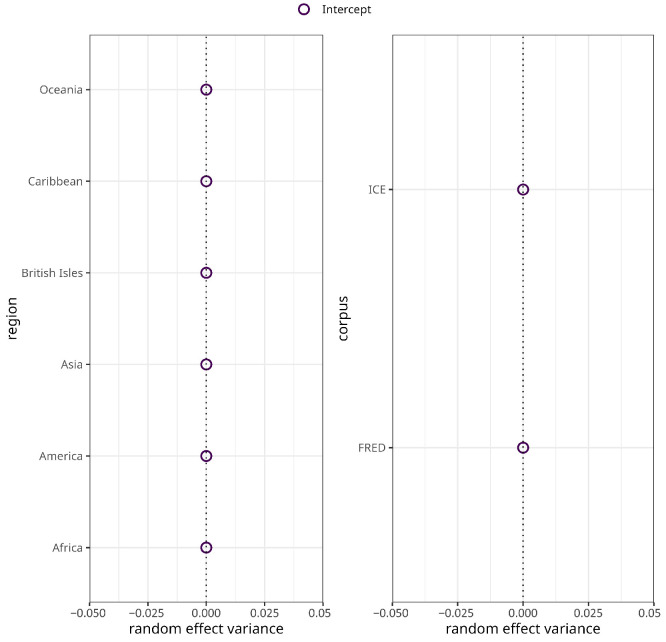
Random effects variances for region (**left panel**) and corpus (**right panel**) in the syntactic complexity model.

The subsequent model for syntactic complexity no longer exhibits harmful collinearity as indicated by variance inflation factors <0.4. However, it still converges with a singular fit. Inspection of the random effects estimates and deviations shows that the estimates and deviations of the varying intercepts for both macro-region and corpus are estimated at zero (Figure 2). To address singularity, the two varying intercepts were consecutively removed until convergence without warnings was reached [52] (p. 266). That said, the resulting model exhibits statistically significant heteroscedasticity (p=0.047), indicating potential model misspecification due to the removal of the varying intercepts. At the same time, however, the data does not support the initially fit random structure so that the model was discarded and is not further discussed.

## 3. Results

### 3.1. Kolmogorov-Based Complexity of English Varieties

This section qualitatively describes the complexity ratios of the English varieties, thereby showcasing the applicability of Kolmogorov complexity as a measure of language complexity.

**Figure 3:**
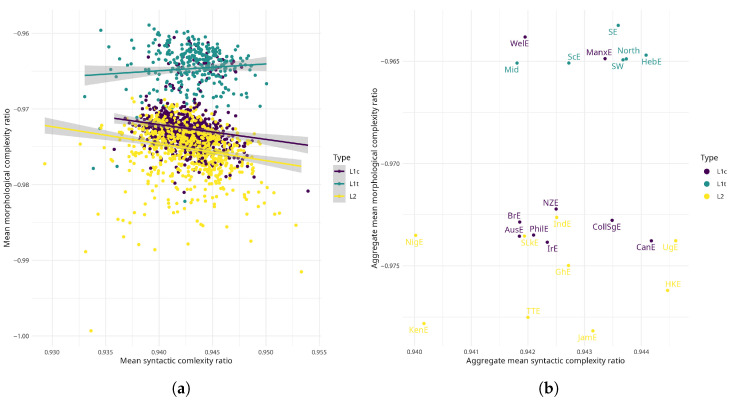
Spoken English varieties by morphological and syntactic complexity and language type. Abscissa indexes increased syntactic complexity; ordinate indexes increased morphological complexity. (**a**) Varieties as individual text files. (**b**) Complexity aggregated by variety.

In terms of morphological complexity, the scatter plots in Figure 3 are largely in line with the theoretically expected varying levels of morphological complexity in different types of English varieties [17,23]. Table A1 lists all varieties by their abbreviations. All of the low-contact L1 varieties cluster together and consistently exhibit higher morphological complexity than the majority of high-contact L1s and all of the indigenised L2 varieties. This means that low-contact L1s generally exhibit the highest amount of word form variation. That said, some of the high-contact L1s (Welsh English and Manx English), which are both shift varieties, are located in the low-contact L1 cluster. The high-contact L1 varieties and some of the Asian L2 varieties (Indian English, Sri Lankan English) form a loose cluster in the middle part of Figure 3b, exhibiting notably less morphological complexity than the traditional L1s. Jamaican and Trinidadian English, together with Kenyan English, are the least morphologically complex varieties in this dataset.

In terms of syntactic complexity, the differences between variety types are not as prominent as in terms of morphological complexity. Most of the varieties exhibit medium syntactic complexity, which means these varieties have roughly equally rigid word order and the same amount of word order patterns. There seems to be a slight tendency for low-contact L1s to exhibit more rigid word order than other variety types. There are also a couple of potential outliers: Ugandan and Hong Kong English are the most syntactically complex varieties, i.e., these varieties exhibit the most rigid word order and least variation in word order patterns. In contrast, Kenyan English and Nigerian English exhibit the least syntactic complexity, which means that they are marked by comparatively more word order patterns compared to the other varieties. Overall, most varieties in FRED and ICE implement the same amount of word order rules—outliers notwithstanding. On an interpretational plane, this could indicate that the varieties/texts in this dataset mostly adhere to the same number of (standard) English word order rules.

Although low-contact L1 and indigenised L2 varieties seem to somewhat trade off morphological for syntactic complexity—indicated by the slightly downwards smoother in Figure 3a—the characteristically observed trade-off between morphological and syntactic complexity [10,22] does not surface in this dataset (Pearson correlation coefficient, r=0.26, p=0.22), neither in terms of Kolmogorov-based syntactic nor morphological complexity.

In short, the pattern that emerges for morphological complexity is in line with the literature, highlighting that complexity as implemented with the compression technique is an adequate measure of morphological complexity. However, there are less prominent differences in syntactic complexity and no clear typological pattern emerges. I hedge earlier in the paper that this is probably due to the type of data analysed. In fact, ICE is well known for sampling “educated” national varieties [53] and it might well be that this is reflected in the amount and distribution of syntactic complexity, i.e., that differences in syntactic complexity are either not measurable with the compression technique or simply not present.

### 3.2. Kolmogorov-Based Complexity and Socio-Demographic Triggers

This section reports the regression results for morphological complexity and socio-demographic triggers. The significance of the fixed effects was assessed with likelihood ratio tests, applying single-term deletions and, for random effects, by individually removing the intercepts and subsequently conducting analyses of variance. Note that all estimates are on a very small scale, which is in correspondence with the small scale of and measured differences between compression ratios.

The likelihood ratio tests for the fixed effects, controlling for corpus and region, show significant differences for population density ($\chi_{\left(1\right)}^{2}=5.25,\;p=0.022$), migration ($\chi_{\left(1\right)}^{2}=8.14$, p=0.004), and contact languages ($\chi_{\left(3\right)}^{2}=13.21,\;p=0.004$). Spread ($\chi_{\left(1\right)}^{2}=3.06$, p=0.08) shows a marginally significant difference, whereas dropping the number of native speakers ($\chi_{\left(1\right)}^{2}=1.19,\;p<0.276$) and proportion of non-native speakers ($\chi_{\left(1\right)}^{2}=2.67,\;p=0.102$) from the model does not result in significant differences between models.

**Figure 4:**
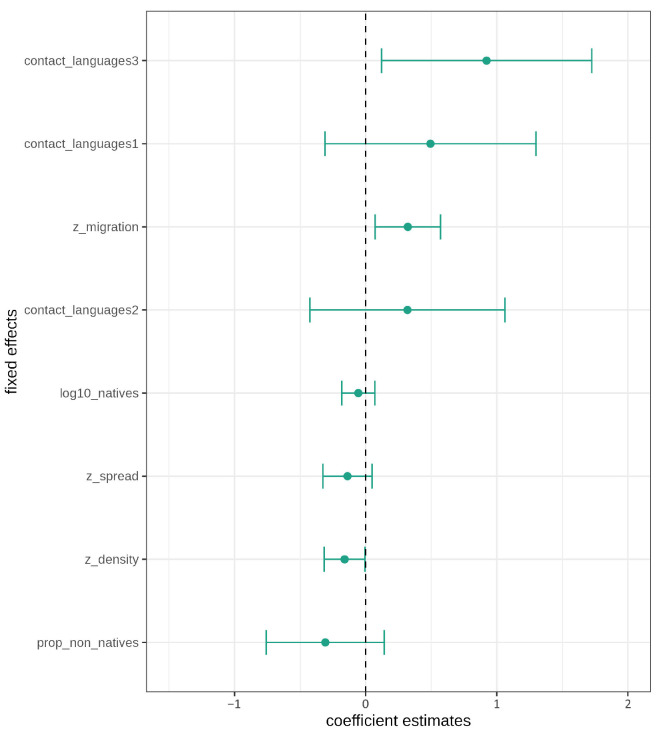
Fixed effects estimates with confidence intervals.

In addition, confidence intervals for the fixed effects were calculated as plotted in Figure 4. The large confidence intervals for contact languages and the proportion of non-native speakers show that there is a lot of uncertainty in estimating these effects. Furthermore, all three levels for contact languages cross the zero boundary and should thus not be considered significant (despite the likelihood ratio tests). Similarly, the confidence intervals for number of native speakers, proportion of non-native speakers, and geographic spread all cross the zero boundary. The non-significance and large uncertainty represented by the large confidence intervals for these triggers is in line with the controversial findings in the typological literature [16,19,20,21]. The only effects not crossing the zero boundary are net migration rate and population density, which thus both have a significant effect on morphological complexity, conditioned on the random effects structure.

According to the contact scenarios outlined in the literature [17,18] and the Linguistic Niche Hypothesis [16], negative effects/coefficient estimates for all socio-demographic triggers are expected, i.e., larger numbers of contact languages and speakers, larger values for spread, population density, and net migration rate, should result in less morphological complexity. In other words, more contact should result in less word form variation. Therefore, the actual effect directions are interesting, independent of the significance of the individual effects. All three levels for contact languages have a positive estimate. The effect of net migration rate is also positive, which means that increased incoming migration does not correlate with decreased morphological complexity but, instead, with increased morphological complexity. Geographic spread and population density both have negative coefficient estimates and thus have a theoretically expected, negative effect on morphological complexity. The effect directions of speaker numbers, both native speakers and proportion of non-native speakers, are negative and therefore also in the theoretically expected direction.

**Figure 5:**
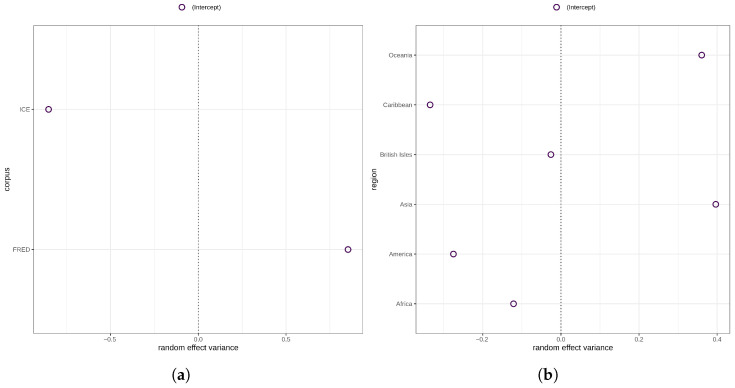
(**a**) Random effects variance for corpus. (**b**) Random effects variance for region.

The major variance in morphological complexity, then, is accounted for by the highly statistically significant random effects for corpus ($\chi_{\left(1\right)}^{2}=24.81,\;p<0$) and region ($\chi_{\left(1\right)}^{2}=9.5$, p=0.002). As a matter of fact, there is ample evidence suggesting that variation in morphological complexity is mainly accounted for by random structures including geography and phylogeny, or effects are not significant when controlling for these random effects [20,21,54]. As can be seen in Figure 5a, the random effects variance for varieties/texts sampled in ICE is negative, whereas the random effects variance for varieties/texts in FRED is positive. This indicates that morphological complexity ratios for ICE varieties are generally lower than for FRED varieties. In terms of region, the picture is more varied. Suffice it to say that the random effects variances for Asia, Oceania, and the Caribbean are the largest, and for the British Isles the smallest.

## 4. Discussion

This paper tested the Linguistic Niche Hypothesis in corpora of English varieties through a usage-based approach and drawing on an information-theoretic, Kolmogorov-based measure of language complexity. The assumption of the Linguistic Niche Hypothesis is basically that language contact is inversely correlated with language complexity such that languages spoken in exoteric communities should exhibit less complex linguistic structures than languages spoken in esoteric communities [16]. Hence, negative effects of all extra-linguistic triggers on morphological complexity were theoretically expected but only partially borne out by the data. Specifically, there are no statistically significant effects for any of the triggers with the exception of a significant positive effect for net migration rate and a significant negative effect for population density. Instead, the varying intercepts for geography and corpus are highly significant. Thus, my results confirm and mirror findings of recent large-scale typological studies which find that effects of speaker data or other socio-demographic triggers on morphosyntactic structures turn out non-significant when controlling for geographic (and genealogical) non-independence [20,21,54].

That said, there are a couple of interesting findings that come with important theoretical implications and which open up new avenues in future complexity research.

First, given the highly controversial and somewhat heated debate on whether the number of native speakers and the proportion of non-native speakers have a negative effect on morphological complexity, a brief discussion of the effect direction of these triggers is warranted. A substantial number of papers report significant negative effects of the number of native speakers (note that in some papers, the number of native speakers is referred to as “population size”) on different morphological and morphosyntactic structures. Among the most influential studies of this type is [16], whose authors analysed the morphological complexity in a typological dataset of more than 2000 languages. Language contact was operationalised in terms of the number of native speakers, geographic spread, and the number of neighbouring contact languages. Their results provide evidence for the influence of socio-demographic triggers on language structure; precisely, larger numbers of native speakers tend to be less morphologically complex and prefer lexical strategies over inflectional ones than languages spoken by smaller numbers of native speakers. Similar evidence is provided by [55] for the negative effect of the number of native speakers on core argument marking, and by [15,56] for the negative effect of the proportion of non-native speakers on lexical diversity and case marking, respectively. At the same time, there is a growing body of evidence suggesting that the number of native speakers and the proportion of non-native speakers—sometimes approximated by *vehicularity*, which is based on a language’s vitality, and assumes that vehicular languages are used for wider exoteric communication [12]—do not negatively correlate with various morphological structures [12,20,21]. In fact, effects of these triggers are sometimes even found to be (weakly) positive [12,21], i.e., larger numbers of native speakers predict higher complexity. Evidence from machine learning further supports these findings: ref. [57] reports that languages spoken by larger numbers of native speakers are associated with higher prediction complexity and, according to a follow-up study [58], are also harder to machine-learn. In this context, it is noteworthy that the effect direction of both the number of native speakers and the proportion of non-native speakers in this paper is negative, albeit non-significant, and therefore theoretically in line with the Linguistic Niche Hypothesis. I believe that this ties in with suggestions that not all morphosyntactic structures and information-theoretic metrics are equally responsive to socio-demographic triggers [14]. Further research in an English-varieties context is therefore clearly needed to test if the effects observed here (1) change direction or (2) become significant when different (and more) data or different complexity measures are modelled. Only incremental and step-wise evidence can provide robust evidence in the spirit of [59] for or against such a complex hypothesis.

Second, the effect for net migration rate is, contrary to expectations, positive. Net migration rate was included as a proxy for both mobility and contact in the sense that higher net migration rates would approximate higher degrees of language contact. However, this idea needs to be revised and the economic implications of high outgoing and high incoming migration rates need to be considered instead. Countries with high incoming migration rates are mostly economically well-off countries; in this dataset, they are represented by low-contact and some high-contact L1 varieties in the British Isles, Australia, and New Zealand, as well as North America. These varieties should and do, in fact, exhibit higher morphological complexity than countries with high outgoing migration rates (here mostly countries in Asia, Africa, and the Caribbean, where indigenised L2 and some of the high-contact L1 varieties are spoken) [23,24]. I tested this relationship post hoc in a simple Kruskal–Wallis rank sum test, which turned out to be highly significant ($\chi_{\left(2\right)}^{2}=14.11$, p<0.001). Thus, against the background of a globalised world, in which English has become a commodity and whose acquisition is an asset for socioeconomic advancement [60] but where access to English is not equally distributed [61], higher net migration rates predict higher morphological complexity. Lower morphological complexity, in this context, is predicted by lower net migration rates and associated with poorer economies and less access to (standard) English (instruction). Net migration rate therefore seems like a promising candidate and a powerful predictor for the complexity of English varieties. Future complexity research should therefore model and also explore other socioeconomic triggers, especially in the landscape of English varieties, which might be a better predictor for complexity variation than speaker data.

Third, population density has a theoretically expected negative and significant effect on morphological complexity, confirming the idea that exoteric speaker communities are also marked by higher population density [38]. The effect of geographic spread was negative, as expected, but also crossed the zero boundary. In contrast, contact languages which are spoken by at least ten percent of the population in a given country or territory turned out to be positive with the largest uncertainty in estimation. This suggests that the current operationalisation of contact languages may not be a good predictor for language contact. An alternative explanation for the positive effect could be that contact languages spoken by at least ten percent of the population in a given territory are not primarily acquired by adult second-language learners but rather acquired earlier, possibly during childhood, as part of speakers’ multilingual repertoires. Such a contact scenario would then be one of intensive long-term contact including child bilingualism. In brief, in such a contact scenario, complexity tends to be fostered or maintained [17,62].

Fourth, the correlations between the socio-demographic triggers deserve a couple of words, although they are, strictly speaking, not the focus of the analysis here. Larger proportions of non-native speakers are very highly positively correlated with the number of contact languages but negatively with net migration rate. So, unsurprisingly, the more non-native speakers there are in a given territory, the more languages are spoken by more than ten percent of the population. Net migration rate moderately positively correlates with the number of native speakers but highly negatively with both the proportion of non-native speakers and contact languages. These high correlations, or the fact that geographic spread and population density do not correlate with any of the other socio-demographic triggers, suggest the existence of interesting geographic sociolinguistic patterns that warrant further exploration.

Finally, on a methodological plane, the paper showed that the compression ratios obtained through the compression technique yield intuitive and expected results in terms of morphological complexity but less so in terms of syntactic complexity. As suggested above, this is likely related to the data used here as previous research using the compression technique with different corpora consistently found that it yields reliable and well-interpretable results which are in line with more traditional measures [10,22]. In general, Kolmogorov complexity as a measure of language complexity is an innovative, inexpensive, and usage-based measure which can be easily applied to naturalistic language corpora. Such a corpus-based approach constitutes an advantage over atlas-based studies because contact-induced structural changes should be captured in such naturalistic data. However, the use of compression ratios to model the role of socio-demographic triggers on complexity posed unexpected challenges and no statistically adequate model for syntactic complexity could be obtained. Possibly, a follow-up study should use larger (and also written) corpora such as the *Corpus of Global Web-Based English* [63].

In sum, this paper mirrors the inconclusive findings in the typological literature in regard to the Linguistic Niche Hypothesis and opens up new avenues for future complexity research. Clearly, more research from a typological and intra-linguistic perspective is needed to solve the conundrum on contact and complexity.

## Data Availability

The original data presented in the study are openly available in github at https://github.com/Morphosyntactic-Variation-in-Englishes/k-complexity. The demographic data can be downloaded from GitHub at https://github.com/Morphosyntactic-Variation-in-Englishes/DOVE, accessed on 11 August 2025.

## Appendix A

**Table A1:** Abbreviations of English varieties.

Variety	Abbreviation
Australian English	AusE
British English	BrE
Canadian English	CanE
Colloquial American English	CollAmE
Colloquial Singapore English	CollSgE
English dialects in the Midlands	Mid
English dialects in the North of England	North
English dialects in the Southeast of England	SE
English dialects in the Southwest of England	SW
Ghanaian English	GhE
Hebridean English	HebE
Indian English	IndE
Irish English	IrE
Jamaican English	JamE
Kenyan English	KenE
Manx English	ManxE
New Zealand English	NZE
Nigerian English	NigE
Philippine English	PhilE
Scottish English	ScE
Sri Lankan English	SLkE
Trinidadian English	TTE
Ugandan English	UgE
Welsh English	WelE

## References

[1] Mufwene S., Coupé C., Pellegrino F. (2017). Complexity in Language: Developmental and Evolutionary Perspectives.

[2] Baechler R., Seiler G. (2016). Complexity, Isolation, and Variation.

[3] Baerman M., Brown D., Corbett G.G. (2015). Understanding and Measuring Morphological Complexity.

[4] Kortmann B., Szmrecsanyi B. (2012). Linguistic Complexity: Second Language Acquisition, Indigenization, Contact.

[5] McWhorter J. (2001). The world’s simplest grammars are creole grammars. Linguist. Typology.

[6] Miestamo M., Miestamo M., Sinnemäki K., Karlsson F. (2008). Grammatical complexity in a cross-linguistic perspective. Language Complexity: Typology, Contact, Change.

[7] Ehret K., Berdicevskis A., Bentz C., Blumenthal-Dramé A. (2023). Measuring language complexity: Challenges and opportunities. Linguist. Vanguard.

[8] Newmeyer F.J., Preston L.B. (2014). Measuring Grammatical Complexity.

[9] Ehret K., Blumenthal-Dramé A., Bentz C., Berdicevskis A. (2021). Meaning and measures: Interpreting and evaluating complexity metrics. Front. Commun..

[10] Ehret K. (2021). An information-theoretic view on language complexity and register variation: Compressing naturalistic corpus data. Corpus Linguist. Linguist. Theory.

[11] Audring J. (2017). Calibrating complexity: How complex is a gender system?. Lang. Sci..

[12] Koplenig A. (2019). Language structure is influenced by the number of speakers but seemingly not by the proportion of non-native speakers. R. Soc. Open Sci..

[13] Di Garbo F., Verkerk A. (2022). A typology of northwestern Bantu gender systems. Linguistics.

[14] Sinnemäki K., Di Garbo F. (2018). Language Structures May Adapt to the Sociolinguistic Environment, but It Matters What and How You Count: A Typological Study of Verbal and Nominal Complexity. Front. Psychol..

[15] Bentz C., Winter B. (2013). Languages with More Second Language Learners Tend to Lose Nominal Case. Lang. Dyn. Change.

[16] Lupyan G., Dale R. (2010). Language Structure Is Partly Determined by Social Structure. PLoS ONE.

[17] Trudgill P. (2011). Sociolinguistic Typology: Social Determinants of Linguistic Complexity.

[18] Wray A., Grace G.W. (2007). The consequences of talking to strangers: Evolutionary corollaries of socio-cultural influences on linguistic form. Lingua.

[19] Kauhanen H., Walkden G., Einhaus S. (2023). Language structure is influenced by the proportion of non-native speakers: A reply to Koplenig (2019). J. Lang. Evol..

[20] Koplenig A. (2024). Still No Evidence for an Effect of the Proportion of Non-Native Speakers on Natural Language Complexity. Entropy.

[21] Shcherbakova O., Michaelis S.M., Haynie H.J., Passmore S., Gast V., Gray R.D., Greenhill S.J., Blasi D.E., Skirgård H. (2023). Societies of strangers do not speak less complex languages. Sci. Adv..

[22] Ehret K., Szmrecsanyi B., Baechler R., Seiler G. (2016). An information-theoretic approach to assess linguistic complexity. Complexity, Isolation, and Variation.

[23] Kortmann B., Szmrecsanyi B., Siebers L., Hoffmann T. (2009). World Englishes between simplification and complexification. World Englishes-Problems, Properties and Prospects: Selected Papers from the 13th IAWE Conference.

[24] Szmrecsanyi B. (2009). Typological parameters of intralingual variability: Grammatical analyticity versus syntheticity in varieties of English. Lang. Var. Change.

[25] Szmrecsanyi B., Kortmann B., Sampson G., Gil D., Trudgill P. (2009). Between simplification and complexification: Non-standard varieties of English around the world. Language Complexity as an Evolving Variable.

[26] R Core Team (2025). R: A Language and Environment for Statistical Computing.

[27] Greenbaum S. (1991). ICE: The international corpus of English. Engl. Today.

[28] Kortmann B., Wagner S., Closs Traugott E., Kortmann B., Kortmann B., Herrmann T., Pietsch L., Wagner S. (2008). The Freiburg English Dialect Project and Corpus (FRED). A Comparative Grammar of British English Dialects.

[29] Du Bois J.W., Chafe W.L., Meyer C., Thompson S.A., Martey N. (2000–2005). Santa Barbara Corpus of Spoken American English, Parts 1–4.

[30] Kortmann B., Lunkenheimer K., Ehret K. (2020). The Electronic World Atlas of Varieties of English.

[31] Comrie B. (1988). Linguistic typology. Annu. Rev. Anthropol..

[32] Ehret K. (2025). Morphosyntactic-Variation-in-Englishes/DOVE: DOVE v1.0 (v1.0).

[33] Cheng L.S.P., Burgess D., Vernooij N., Solís-Barroso C., McDermott A., Namboodiripad S. (2021). The Problematic Concept of Native Speaker in Psycholinguistics: Replacing Vague and Harmful Terminology With Inclusive and Accurate Measures. Front. Psychol..

[34] Ehret K. (2025). How to obtain speaker numbers for English varieties around the world: Theoretical concepts, challenges and estimations. Engl. World-Wide.

[35] Berdicevskis A., Semenuks A., Arkadiev P., Gardani F. (2020). Different trajectories of morphological overspecification and irregularity under imperfect language learning. The Complexities of Morphology.

[36] Bentz C., Berdicevskis A. Learning pressures reduce morphological complexity: Linking corpus, computational and experimental evidence. Proceedings of the 26th International Conference on Computational Linguistics (COLING 2016).

[37] Atkinson M., Smith K., Kirby S. (2018). Adult Learning and Language Simplification. Cogn. Sci..

[38] Chen S., Gil D., Gaponov S., Reifegerste J., Yuditha T., Tatarinova T., Progovac L., Benítez-Burraco A. (2024). Linguistic correlates of societal variation: A quantitative analysis. PLoS ONE.

[39] Thomason S.G., Kaufman T. (1991). Language Contact, Creolization, and Genetic Linguistics.

[40] Lunkenheimer K. (2012). Typological profile: L2 varieties. The Mouton World Atlas of Variation in English.

[41] Kortmann B., Wolk C. (2012). Morphosyntactic variation in the anglophone world: A global perspective. The Mouton World Atlas of Variation in English.

[42] Szmrecsanyi B. (2012). Typological profile: L1 varieties. The Mouton World Atlas of Variation in English.

[43] Kolmogorov A.N. (1965). Three Approaches to the Quantitative Definition of Information. Probl. Peredachi Informatsii.

[44] Kolmogorov A. (1963). On Tables of Random Numbers. Sankhya.

[45] Juola P. (1998). Measuring linguistic complexity: The morphological tier. J. Quant. Linguist..

[46] Juola P., Miestamo M., Sinnemäki K., Karlsson F. (2008). Assessing linguistic complexity. Language Complexity: Typology, Contact, Change.

[47] Li M., Vitányi P.M.B. (1997). An Introduction to Kolmogorov Complexity and Its Applications.

[48] Li M., Chen X., Li X., Ma B., Vitányi P.M.B. (2004). The similarity metric. IEEE Trans. Inf. Theory.

[49] Ziv J., Lempel A. (1977). A universal algorithm for sequential data compression. IEEE Trans. Inf. Theory.

[50] Ehret K. (2017). An Information-Theoretic Approach to Language Complexity: Variation in Naturalistic Corpora. Ph.D. Thesis.

[51] Bates D., Mächler M., Bolker B., Walker S. (2015). Fitting Linear Mixed-Effects Models Using lme4. J. Stat. Softw..

[52] Barr D.J., Levy R., Scheepers C., Tily H.J. (2013). Random effects structure for confirmatory hypothesis testing: Keep it maximal. J. Mem. Lang..

[53] Greenbaum S. (1990). Standard English and the international corpus of English. World Englishes.

[54] Guzmán Naranjo M., Becker L. (2022). Statistical bias control in typology. Linguist. Typology.

[55] Sinnemäki K., Sampson G., Gil D., Trudgill P. (2009). Complexity in core argument marking and population size. Language Complexity as an Evolving Variable.

[56] Bentz C., Verkerk A., Kiela D., Hill F., Buttery P. (2015). Adaptive Communication: Languages with More Non-Native Speakers Tend to Have Fewer Word Forms. PLoS ONE.

[57] Koplenig A., Wolfer S., Meyer P. (2023). A large quantitative analysis of written language challenges the idea that all languages are equally complex. Sci. Rep..

[58] Koplenig A., Wolfer S. (2023). Languages with more speakers tend to be harder to (machine-) learn. Sci. Rep..

[59] Roberts S.G. (2018). Robust, causal, and incremental approaches to investigating linguistic adaptation. Front. Psychol..

[60] Cameron D., Nevalainen T., Traugott Closs E. (2012). The commodification of language: English as a global commodity. The Oxford Handbook of the History of English.

[61] Tupas R. (2019). Unequal Englishes as a sociolinguistics of globalization. J. Engl. Stud. Comp. Lit..

[62] Nichols J., Sampson G., Gil D., Trudgill P. (2009). Linguistic complexity: A comprehensive definition and survey. Language Complexity as an Evolving Variable.

[63] Davies M. (2013). Corpus of Global Web-Based English. https://www.english-corpora.org/glowbe/.

